# A self-controlled case series to assess the effectiveness of beta blockers for heart failure in reducing hospitalisations in the elderly

**DOI:** 10.1186/1471-2288-11-106

**Published:** 2011-07-18

**Authors:** Emmae N Ramsay, Elizabeth E Roughead, Ben Ewald, Nicole L Pratt, Philip Ryan

**Affiliations:** 1 Data Management and Analysis Centre, Discipline of Public Health, University of Adelaide, South Australia, 5000, Australia; 2 School of Pharmacy and Medical Sciences, Quality Use of Medicines and Pharmacy Research Centre; Sansom Institute, University of South Australia, South Australia, 5000, Australia; 3 School of Medicine and Public Health, Faculty of Health, University of Newcastle, NSW 2308 Australia

## Abstract

**Background:**

To determine the suitability of using the self-controlled case series design to assess improvements in health outcomes using the effectiveness of beta blockers for heart failure in reducing hospitalisations as the example.

**Methods:**

The Australian Government Department of Veterans' Affairs administrative claims database was used to undertake a self-controlled case-series in elderly patients aged 65 years or over to compare the risk of a heart failure hospitalisation during periods of being exposed and unexposed to a beta blocker. Two studies, the first using a one year period and the second using a four year period were undertaken to determine if the estimates varied due to changes in severity of heart failure over time.

**Results:**

In the one year period, 3,450 patients and in the four year period, 12, 682 patients had at least one hospitalisation for heart failure. The one year period showed a non-significant decrease in hospitalisations for heart failure 4-8 months after starting beta-blockers, (RR, 0.76; 95% CI (0.57-1.02)) and a significant decrease in the 8-12 months post-initiation of a beta blocker for heart failure (RR, 0.62; 95% CI (0.39, 0.99)). For the four year study there was an increased risk of hospitalisation less than eight months post-initiation and significant but smaller decrease in the 8-12 month window (RR, 0.90; 95% CI (0.82, 0.98)).

**Conclusions:**

The results of the one year observation period are similar to those observed in randomised clinical trials indicating that the self-controlled case-series method can be successfully applied to assess health outcomes. However, the result appears sensitive to the study periods used and further research to understand the appropriate applications of this method in pharmacoepidemiology is still required. The results also illustrate the benefits of extending beta blocker utilisation to the older age group of heart failure patients in which their use is common but the evidence is sparse.

## Background

Administrative claims databases are being used more widely around the world for research[1], in particular, in pharmacoepidemiology. Research to assess the practical viability of study designs using administrative data in a variety of contexts is imperative so that policy makers and health professionals can be more confident in the conclusions that are made using these data sources.

In pharmacoepidemiological studies it can be difficult to measure and control for the differences between patients who were prescribed and not prescribed a medicine of interest,[2] due to important potential confounders not being available in the data for use by researchers[1,3]. Inadequate control of differences between groups may lead to confounding in assessing the association between an exposure and outcome of interest[1,3]. Traditional observational study designs such as case-control and cohort studies cannot adjust for unknown, unmeasured or poorly measured confounders[4]. The self-controlled case series method is gaining popularity in pharmacoepidemiology as an alternative study design to cohort and case-control designs. The main advantage of this method is that it minimises confounding due to its within-person design, where the patient acts as their own control [5,6]. The within person design controls implicitly for fixed known and unknown confounders that do not vary over time, such as genetic and socio-economic factors. Other time varying confounders such as age can be adjusted within the model [5,6].

The self controlled case series design includes only those individuals who have had an outcome of interest. A comparison is made between the rate of events during periods of exposure and non-exposure to the drug of interest. Confounding by indication can also be assessed and controlled for in this method through the use of pre-exposure risk periods. Confounding by indication is present if patient characteristics alter the likelihood of being prescribed a medicine and are at the same time related to the probability of an outcome[7].

The self controlled case-series design has been used to assess the adverse events of medicines[2,8-14] and has been identified as a potential tool for post-marketing surveillance of medicines[12]. To date, this method has not been used to assess the effectiveness of medicines. In this study we used the example of beta-blockers for heart failure to assess whether the self-controlled case series method can be applied to study the effectiveness of medicines. The effectiveness of beta-blockers in heart failure was chosen as a test case as there is evidence from randomised controlled trials that beta blockers reduce hospitalisations for heart failure[15] and the outcome of reduced hospitalisations has been observed in short term trials of twelve months or less.

Randomised controlled trial evidence has led to beta blockers being recommended in current clinical guidelines as first line agents for patients with symptomatic or advanced chronic heart failure [16]. Clinical trials of beta blockers for heart failure predominately recruited participants who were on average in their early sixties, which is about 10-15 years younger than those living in the community with heart failure[17]. Thus evidence for the efficacy of beta-blockers for heart failure in the elderly is limited. Few observational studies have been conducted in the elderly with heart failure and confounding by indication being identified as possible limitations of these studies[18].

The objective of this study was to determine the suitability of using the self-controlled case series design to assess improvements in health outcomes using the example of effectiveness of beta-blockers for heart failure in reducing the risk of hospitalisation for heart failure.

## Methods

The data source for this study was the Australian Department of Veterans' Affairs (DVA) administrative claims databases. This database is not publicly available and was provided by the Department of Veterans Affairs as part of the delivery of the Veterans' Medicines Advice and Therapeutics Education Services (Veterans' MATES) project. DVA claims data contain records of prescription medicines dispensed under the Repatriation Pharmaceutical Benefits Scheme, medical and allied health services and hospital admissions provided to veterans for whom DVA pays a subsidy. The treatment population is approximately 310 000 veterans, and there are approximately 100 million pharmacy records, 200 million medical and allied health service records and over 6 million hospital admission records. A client file is maintained by DVA which includes data on sex, date of birth, date of death and family status.

A self controlled case series study was conducted. Since heart failure is a disease that is not stable over time and can cause patients to deteriorate quickly, two studies with differing follow-up times were undertaken. The one year period was chosen as this is similar to the randomised controlled trial periods and is also likely to be a period where heart failure is more stable. The four year period was chosen to determine if the method can assess effectiveness over a longer time period. The two observation periods used were a one year period starting 1^st ^July 2005 and a four year period starting 1^st ^July 2002 and with both studies ending on the 30^th ^June 2006. All veterans who had a hospitalisation with a primary diagnosis of heart failure (ICD-10 I500, I501, I509) during the observation period, who were aged 65 years or over at study entry and who were eligible for all health services subsided by the Department of Veterans' Affairs were included. For these veterans, prescription data on beta blockers for heart failure (bisoprolol, carvedilol, and metoprolol succinate) were identified. These beta blockers are only available under a prior authorization process where the prescribing physician must indicate at the time of prescription that the patients have the diagnosis of heart failure. Analysis of the data showed that 75% of veterans returned for a refill prescription within 36 days, so the duration of each prescription was assumed to be 36 days.

Only incident users of beta blockers for heart failure were included. Those who had been dispensed a beta blocker for heart failure in the 12 months prior to study start were excluded. Heart failure is a progressive disease that can cause an increase in the risk of a hospital admission for heart failure over time regardless of treatment. Since this study includes long indefinite risk periods it maybe more prone to possible confounding between age and exposure effects[6]. This possible confounding is reduced in this study by the inclusion of veterans who had a hospitalisation for heart failure but were not exposed to a beta blocker [6]. These patients were expected to contribute information on the impact of age on the risk of the outcome.

**Figure 1:**
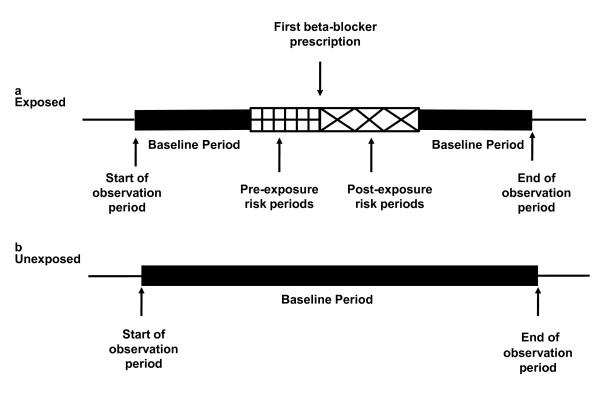
**A graphical representation of the self-controlled case-series study design for patients (a) exposed to a beta-blocker (b) unexposed to a beta-blocker**.

Person time was divided into risk periods; 1 day-2 weeks, 2-4 weeks, 1-4 months, 4-8 months, 8-12 months and for the four year study period >12 months post beta blocker initiation. Four consecutive 2 week pre-exposure risk periods were included prior to beta blocker initiation to remove the effect of confounding by indication from the unexposed time. The actual day of the prescription was excluded because in cases where the heart failure hospitalisation occurred on the same day it was not possible to determine which occurred first. All remaining time was considered unexposed to a beta-blocker for heart failure, including all the follow-up time of an unexposed individual and was used as the baseline comparison period (Figure 1).

In each risk period, the cumulative number of hospitalisations was divided by the person years at risk and these were compared to the baseline period. If a veteran was re-hospitalised within 30 days, the subsequent hospitalisation(s) were excluded as they were considered to be related, and part of the same episode [6]. Rate ratios were calculated using conditional Poisson regression, with results presented as adjusted rate ratios and 95% confidence intervals. While the self-controlled case series method controls implicitly for fixed covariates, there may be important confounders/covariates that change over time within person that should be controlled for in the analysis. Therefore, adjustment was made for age at heart failure hospitalisation[8], study month and time-varying covariates which were assessed quarterly; co-morbidities using the Australian adaption of Rx-Risk-V[19], number of prescribers, admission into aged care and prescription of frusemide, ACE/A2RB, digoxin and aldosterone antagonists. Sensitivity analyses were undertaken excluding non-exposed patients and the one year study was conducted over different calendar periods to assess the effects of calendar time. All analyses were performed using SAS version 9.12 (SAS Institute, Cary, NC).

## Results

In the one year observation period there were 3,450 veterans who had at least one hospitalisation for heart failure and of those 645 (19%) were initiated on a beta blocker for heart failure. There were 12,682 patients who had at least one hospitalisation for heart failure in the four year study period, with 3,276 (26%) initiated on a beta blocker for heart failure. Table 1 contains the characteristics of the study population. For both observation periods the length of follow-up and age at first exposure was similar for the exposed and unexposed groups.

**Table 1:** Characteristics of study subjects by period of observation

Exposure Status	Number of Subjects	Follow-up Years*	Duration of exposure*	Age at first hospitalisation*	Age at first exposure*
**1 Year observation period**					
Exposed	645	0.5(0.3,0.7)	0.3(0.1,0.6)	81(79,84)	81(78,84)
Unexposed	2805	0.5(0.3,0.8)		82(79,86)	
**4 Year observation period**					
Exposed	3276	2.0(1.1,3.0)	0.8(0.2,1.8)	83(80,86)	81(78,84)
Unexposed	9406	1.7(1.0,3.0)		83(80,87)	

*Median (q1, q3)

The results of the one year observation showed that in the post-exposure risk periods there was a significant increased risk before 2 weeks, a non-significant decrease 4-8 months and significant decrease in hospitalisations for heart failure 8-12 months post-initiation of beta blocker compared to baseline. In the pre-exposure risk periods there was a significant increase less than 6 weeks and a non-significant difference 6-8 weeks (table 2).

**Table 2:** Case-series adjusted analysis for 1 year observation period

Risk Periods	Number of Hospitalisations	Person-Years	Adjusted age and study month only RR(95% CI)	Adjusted* RR(95% CI)
**Baseline unexposed Period**				
Unexposed	3275	2660	1.00 (1.00 - 1.00)	1.00 (1.00 - 1.00)
**Pre-exposure to a beta blocker for heart failure**				
6-8 weeks	18	22	1.37 (0.93 - 2.01)	1.29 (0.88 - 1.89)
4-6 weeks	52	23	3.74 (2.90 - 4.82)	3.47 (2.70 - 4.46)
2-4 weeks	87	24	5.85 (4.72 - 7.26)	4.97 (4.02 - 6.16)
1 day-2 weeks	255	25	15.98 (13.51 - 18.91)	12.72 (10.74 - 15.07)
**Post-exposure to a beta blocker for heart failure**				
1 day-2 weeks	46	25	2.85 (2.18 - 3.72)	2.21 (1.70 - 2.88)
2-4 weeks	25	23	1.63 (1.16 - 2.29)	1.29 (0.92 - 1.80)
1-4 months	94	107	1.34 (1.08 - 1.67)	1.18 (0.94 - 1.47)
4-8 months	46	76	0.78 (0.58 - 1.04)	0.76 (0.57 - 1.02)
8-12 months	15	24	0.66 (0.42 - 1.05)	0.62 (0.39 - 0.99)

*Adjusted for age at heart failure hospitalisation[8], study month and time-varying covariates which were assessed quarterly; co-morbidities using the Australian adaption of Rx-Risk-V[19], number of prescribers, admission into aged care and prescription of frusemide, ACE/A2RB, digoxin and aldosterone antagonists

For the four year observation period, the post-exposure risk periods indicated that before eight months post initiation of a beta blocker there was an increased risk of hospitalisation for heart failure and a significant decrease greater than eight months post initiation compared to the baseline period. In all pre-exposure risk periods there was a statistically significant increased risk of having a hospitalisation for heart failure compared to the baseline period (table 3).

**Table 3:** Case-series analysis for 4 year observation period

Risk Periods	Number of Hospitalisations	Person-Years	Adjusted age and study month only RR(95% CI)	Adjusted* RR(95% CI)
**Baseline unexposed Period**				
Unexposed	13541	34944	1.00 (1.00 - 1.00)	1.00 (1.00 - 1.00)
**Pre-exposure to a beta blocker for heart failure**				
6-8 weeks	137	147	2.22 (2.00 - 2.45)	2.24 (2.02 - 2.48)
4-6 weeks	237	148	3.79 (3.50 - 4.11)	3.82 (3.52 - 4.14)
2-4 weeks	380	149	5.93 (5.54 - 6.34)	5.92 (5.54 - 6.34)
1 day-2 weeks	872	150	13.04 (12.38 - 13.73)	12.95 (12.29 - 13.63)
**Post-exposure to a beta blocker for heart failure**				
1 day-2 weeks	190	149	2.75 (2.52 - 3.01)	2.74 (2.51 - 3.00)
2-4 weeks	156	145	2.27 (2.06 - 2.50)	2.27 (2.06 - 2.50)
1-4 months	488	734	1.71 (1.61 - 1.82)	1.68 (1.58 - 1.79)
4-8 months	322	685	1.21 (1.12 - 1.30)	1.18 (1.10 - 1.27)
8-12 months	212	535	0.93 (0.85 - 1.02)	0.90 (0.82 - 0.98)
>12 months	458	1358	0.65 (0.60 - 0.70)	0.61 (0.57 - 0.66)

*Adjusted for age at heart failure hospitalisation[8], study month and time-varying covariates which were assessed quarterly; co-morbidities using the Australian adaption of Rx-Risk-V[19], number of prescribers, admission into aged care and prescription of frusemide, ACE/A2RB, digoxin and aldosterone antagonists

In both studies, the risk estimates for the models adjusted for age and study month were comparable to those observed in the models adjusted for all confounders (tables 2, 3). The results of all one year sensitivity analyses, which are not presented, showed the same trends as those observed in the one year period that is shown.

## Discussion

This study using a self controlled case-series design, found variable results from the two different time periods, however, results from both studies suggest that long-term treatment with beta-blockers for heart failure is associated with a significant reduction in the risk of hospitalisation due to heart failure. The results from the one year study period are comparable with those seen in a meta-analysis[15] which found a reduction in hospital admissions for heart failure for patients randomised to receive beta blockers compared to controls (OR, 0.63; 95% CI (0.56, 0.71) P < 0.0001)[15]. The average length of treatment for the studies included in the meta-analyses was 11 months[15]. This compares with the results seen in the later risk periods of our one year study period where there was a non-significant decrease in heart failure hospitalisations 4-8 months (RR, 0.76; 95% CI (0.57-1.02)) and a significant decrease >8 months, post-initiation of a beta blocker compared to baseline (RR, 0.62; 95% CI (0.39, 0.99)). The results of the longer four year study showed a statistically significant decrease in hospital admissions only greater than 8 months post initiation of a beta blocker (8-12 months: RR, 0.90; 95% CI (0.82, 0.98): >12 months: RR, 0.61; 95% CI (0.57, 0.66)) and by contrast an increased risk in the 4 to 8 month period post initiation of a beta-blocker. Compared to the 1 year results, the decrease was seen later and the comparable risk estimates approximately a third smaller.

Our study shows that the length of observation period chosen for the self-controlled case series is an important factor to consider when utilising this method for effectiveness research. In the four year study beta blockers appear to be less effective when compared to the one year period. This may be due to several factors including a difference in the proportion of the unexposed person-years for exposed subjects prior to the prescription of a beta blocker in the 4 year compared to the one year period (93% and 85% respectively). Prior to exposure to beta-blockers, patients are likely to be healthier as initiation of beta blockers usually occurs when a patient's heart failure cannot be controlled with angiotensin-converting enzyme inhibitors alone or their symptoms have progressed to advanced heart failure[16]. Due to heart failure being a disease with a short life expectancy and an increased risk of re-hospitalisation over time, it is likely that the four year results are less reliable.

For both our study periods, we found that the risk of hospitalisation for heart failure was increased in the first weeks following treatment initiation. This finding was not unexpected as an RCT assessing carvedilol found that initiation of beta blockers caused worsening heart failure in 5.9% of study participants during the two week run-in period[20].

The self-controlled case series method was designed to evaluate the association between a transient exposure and an acute event[5,6]. However, it may be applied to non-acute events that occur long after initial exposure[5,6]. When long risk periods are used, which is the case in this example confounding between age and exposure effects can be more pronounced. Simulation studies have found that inclusion of an unexposed group eliminates this[5,6]. Therefore, in our studies we have included an unexposed group.

For both study lengths, the unadjusted and adjusted results were similar which is to be expected as the main advantage of the self-controlled case series method is its within-subject design that minimises confounding by controlling implicitly for fixed known and unknown confounders [5,6].

The self-controlled case-series method has three key assumptions[5,6]. The first assumption states that recurrent outcome events must be independent, that is, the occurrence of one event must not alter the probability of a subsequent event occurring [5,6]. In our example, once a person has had a hospitalisation for heart failure their short-term risk of experiencing another may be increased. Therefore, hospitalisations may cluster within independent episodes, usually within 30 days of admission. One strategy to deal with this was to only include the first hospital admission of each episode[6]. The second assumption is that the occurrence of an event must not alter the probability of subsequent exposures[5,6]. This assumption is rarely, if ever, met when assessing the effectiveness of medicines to prevent hospitalisations. This is because medicines are routinely initiated during a hospital stay. This is a form of confounding by indication. This problem can be assessed and controlled for through the use of pre-exposure risk periods. If pre-exposure risk periods are not removed from the baseline unexposed time, then the relative risk will be biased towards the null[6], as the risk in the unexposed period would be inflated. The final assumption is that the occurrence of the event of interest must not censor or affect the observation period[5,6]. This assumption would frequently be violated when the outcome event is a hospital admission. This is due to the fact that the hospitalisation will likely increase the probability of death. However, Farrington et al[21], have shown that this method may be robust to failure of this assumption.

One of the main strengths of this study was the use of the self-controlled case series design which has the ability to overcome confounding issues that can affect cohort and case-control studies, in particular confounding by indication. Pre-exposure risk periods for both studies showed significant increases in the rate of hospitalisations prior to being exposed to a beta blocker for heart failure, demonstrating that the likelihood of being prescribed a beta blocker increases after an event of a heart failure hospitalisation, confirming that confounding by indication is present.

## Conclusions

The self-controlled case series method has not been used in pharmacoepidemiological research, to address questions about the effectiveness of medicines. Therefore, the findings of this study are important in gaining knowledge on the usefulness of this study design for this purpose. This study demonstrated that the choice of observation period is important particularly when the underlying disease under study is not stable over time. Changes in a patient's disease severity make it difficult to adequately assess the effectiveness of a medicine over a long period of time. Further research is necessary to develop guidelines for the appropriate use of the self-controlled case series design and to understand when it can be applied most effectively. The results of this study when we restricted to a one year observation period were consistent with those observed in randomised clinical trials indicating that the self-controlled case-series method can be successfully applied to assess health outcomes using a claims database. The results also illustrate the benefits of extending beta blocker utilisation to the older age group of heart failure patients in which their use is common but the evidence is sparse.

## Abbreviations

(DVA): Department of Veterans' Affairs; (RCT): randomised clinical trial.

## Competing interests

The authors declare that they have no competing interests.

## Authors' contributions

ENR participated in the design of the study, performed the statistical analysis, interpreted the data and drafted the manuscript. EER participated in the design, interpretation and to draft the manuscript. BE participated in the design, interpretation and to draft the manuscript. NP participated in the design, interpretation and to draft the manuscript. PR participated in the design and interpretation of the data. All authors read and approved the final manuscript.

## Pre-publication history

The pre-publication history for this paper can be accessed here:

http://www.biomedcentral.com/1471-2288/11/106/prepub
